# Trends of the Dengue Serotype-4 Circulation with Epidemiological, Phylogenetic, and Entomological Insights in Lao PDR between 2015 and 2019

**DOI:** 10.3390/pathogens9090728

**Published:** 2020-09-03

**Authors:** Elodie Calvez, Virginie Pommelet, Somphavanh Somlor, Julien Pompon, Souksakhone Viengphouthong, Phaithong Bounmany, Thep Aksone Chindavong, Thonglakhone Xaybounsou, Phoyphaylinh Prasayasith, Sitsana Keosenhom, Paul T. Brey, Olivier Telle, Marc Choisy, Sébastien Marcombe, Marc Grandadam

**Affiliations:** 1 Arbovirus and Emerging Viral Diseases Laboratory, Institut Pasteur du Lao PDR, Vientiane 01030, Laos; s.somlor@pasteur.la (S.S.); s.viengphouthong@pasteur.la (S.V.); p.bounmany@pasteur.la (P.B.); t.chindavong@pasteur.la (T.A.C.); t.xaybounsou@pasteur.la (T.X.); p.prasayasith@pasteur.la (P.P.); s.keosenhom@pasteur.la (S.K.); marc.grandadam@def.gouv.fr (M.G.); 2 Epidemiology Unit, Institut Pasteur du Lao PDR, Vientiane 01030, Laos; v.pommelet@pasteur.la; 3 Department of Emerging Infectious Diseases, Duke-NUS Medical School, Singapore 169857, Singapore; julien.pompon@ird.fr; 4 MIVEGEC, University of Montpellier, CNRS, IRD, 34394 Montpellier, France; 5 Medical Entomology and Vector Borne Disease Unit, Institut Pasteur du Lao PDR, Vientiane 01030, Laos; p.brey@pasteur.la (P.T.B.); s.marcombe@pasteur.la (S.M.); 6 Centre de Sciences Humaines (CHS), Centre National de la Recherche Scientifique (CNRS), Delhi 110001, India; telle.olivier@gmail.com; 7 Center for Policy Research (CPR), Delhi 110001, India; 8 Nuffield Department of Medicine, University of Oxford, Oxford OX3 7LF, UK; mchoisy@oucru.org; 9 Oxford University Clinical Research Unit, Ho Chi Minh City 700000, Vietnam; 10 Institut de Recherche Biomédicale des Armées, 91220 Brétigny-sur-Orge, France

**Keywords:** dengue, DENV-4, epidemic, Lao PDR, phylogeny, *Aedes* vectors

## Abstract

Dengue outbreaks have regularly been recorded in Lao People’s Democratic Republic (PDR) since the first detection of the disease in 1979. In 2012, an integrated arbovirus surveillance network was set up in Lao PDR and an entomological surveillance has been implemented since 2016 in Vientiane Capital. Here, we report a study combining epidemiological, phylogenetic, and entomological analyzes during the largest DENV-4 epidemic ever recorded in Lao PDR (2015–2019). Strikingly, from 2015 to 2019, we reported the DENV-4 emergence and spread at the country level after two large epidemics predominated by DENV-3 and DENV-1, respectively, in 2012–2013 and 2015. Our data revealed a significant difference in the median age of the patient infected by DENV-4 compared to the other serotypes. Phylogenetic analysis demonstrated the circulation of DENV-4 Genotype I at the country level since at least 2013. The entomological surveillance showed a predominance of *Aedes* *aegypti* compared to *Aedes* *albopictus* and high abundance of these vectors in dry and rainy seasons between 2016 and 2019, in Vientiane Capital. Overall, these results emphasized the importance of an integrated approach to evaluate factors, which could impact the circulation and the epidemiological profile of dengue viruses, especially in endemic countries like Lao PDR.

## 1. Introduction

In the context of globalization of trade and travel, the arboviruses’ epidemiology profiles have changed and their expansion is in constant progression [1]. Dengue fever is the most prevalent human arboviral disease in the world. Recent World Health Organization (WHO) statistics revealed an increase of the number of dengue cases reported from 505,430 cases in 2000 to 4.2 million in 2019, among which 70% of the burden is supported by Asia, and a modelling study estimated that 390 million people are infected by dengue virus per year [2,3,4]. Even if these data should be interpreted cautiously, due to changes in declaration systems and the increased number of contributing countries, they do reflect an alarming evolution of dengue virus (DENV) epidemiology. In 2017, WHO estimated that, every year, more than 500,000 people, including a high proportion of children, experience severe clinical presentations that require hospitalization. The number of fatal dengue cases has increased from 960 in 2000 to 4032 in 2015 [4].

Dengue fever is due to an infection of one of the four DENV serotypes transmitted to humans through the bite of an infected female mosquito of the *Aedes* genus [5]. In Lao People’s Democratic Republic (Lao PDR), the main vector in urban areas is *Aedes aegypti* [6], whereas *Aedes albopictus* is considered a secondary vector, specifically in suburban, rural, and forested areas [5,7,8].

DENV are single-stranded, positive-sense RNA viruses belonging to the *Flavivirus* genus, *Flaviviridae* family. Antigenic studies identified four distinct serotypes (DENV-1 to 4) [9]. Genetic studies confirmed the segregation of DENV strains into four main groups matching with the serotypes, but also into genotype subdivisions that are often characteristic of the geographical origin of the viral strains [10,11,12,13,14]. Previous genetic studies have suggested that the four DENV serotypes emerged from sylvatic cycles in Asia [11,15,16]. Dengue outbreaks caused by the four serotypes have regularly been recorded in Asia over the last decades [17]. DENV-4 was identified for the first time in the Philippines and in Thailand in 1953 [17]. Since then, DENV-4 has been reported yearly in the Indochinese peninsula, but with a global burden significantly lower compared to the three other DENV serotypes [17]. From a genetic point of view, DENV-4 isolates segregate in four different genotypes: Genotype I (Asia) is frequently reported in South East Asia [18]; Genotype II (Asia, South Pacific, and South America); Genotype III (Thailand); and Genotype IV (sylvatic strains from Malaysia) [11,16,19,20,21].

Lao PDR is a land-locked country located in the middle of the Indochinese peninsula, surrounded by China (North), Cambodia (South), Thailand and Myanmar (West), and Vietnam (East). Lao PDR was a least-developed country of 7 million people in 2018, among which nearly 40% are under 20 years old (WHO and www.lsb.gov.la). The country is divided into 18 provinces. Since the first report of dengue hemorrhagic fever (DHF) cases in Lao PDR in 1979, DENV outbreaks have been regularly declared by the country and dengue fever has become a major national public health problem [22,23,24]. All four dengue serotypes now circulate in rural and urban areas in Lao PDR [22,23,25,26,27]. In 2008, a DENV-1 epidemic was described only in the North-West part of the country [28]. Then, in 2013, an extensive dengue outbreak with a predominance of DENV-3 caused 48,772 cases and 95 deaths at the country scale [29].

Interestingly, DENV-4 has been rarely detected in Lao PDR. Previous studies reported a lower prevalence of this serotype compared to the others in 1990 in Vientiane Capital and in other provinces in 2008–2009 [22,29,30]. Only sporadic cases have been detected in villages located around Vientiane Capital in 2006–2007 [30]. In 2012, an integrated arbovirus surveillance system, involving virologists and medical entomologists, was set up in Vientiane Capital by the Institut Pasteur du Laos and was gradually extended to different provinces in Lao PDR for the detection and monitoring of arbovirus epidemics [19,20,31]. This surveillance system detected DENV-4 transmission in 2013 in Vientiane Capital’s suburban areas and subsequently its spread into the different districts of the city and then throughout the country.

This study aims to describe the DENV-4 switch from an endemic-sporadic circulation to a country-wide epidemic in Lao PDR in the context of multiple DENV serotypes co-circulation. A retrospective epidemiological study was conducted in Vientiane Capital to characterize the impact of the disease burden on the population. Furthermore, an entomological study was implemented in Vientiane Capital to estimate the population dynamics of the vectors at different time points between 2016 and 2019.

## 2. Results

### 2.1. Dengue Surveillance Activity between 2012 and 2019.

From 2012 to 2019, 15,152 samples were collected through the arbovirus surveillance system in 15 of the 18 provinces of Lao PDR (Table S1; Figure S1).

Of the 15,152 samples tested, 8771 (57.9%) were confirmed for a DENV infection by means of real-time RT-PCR and/or NS1 antigen detection. Among the confirmed cases, 8315 (54.9%) were found positives for DENV by RT-PCR and 448 (5%) cases were confirmed by NS1 antigen detection in plasma or in urine samples. The DENV serotypes were determined by real-time RT-PCR or sequencing after a viral culture for 3616 cases (43.5%).

**Figure 1:**
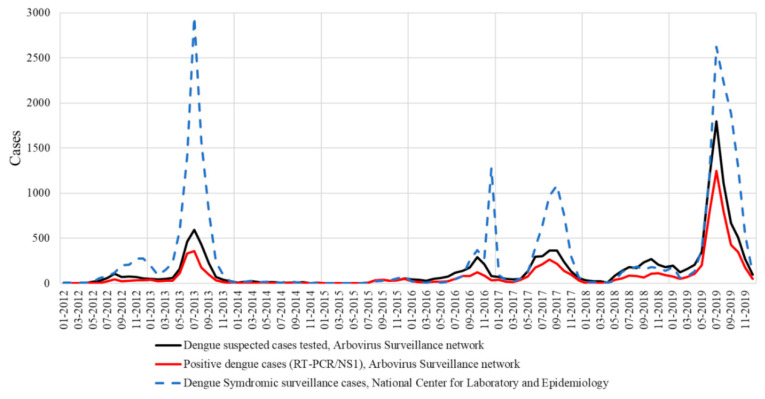
National syndromic surveillance system versus laboratory-based survey in Lao PDR.

**Figure 2:**
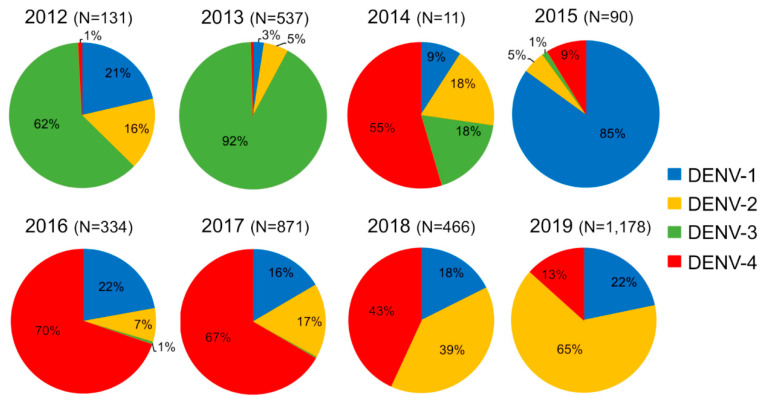
Dengue serotype distribution between 2012 and 2019 from samples collected by the Institut Pasteur du Laos arbovirus surveillance network. In parentheses, number of samples tested per year.

Overall, 11,858 samples (78.4%) were collected in Vientiane Capital, notwithstanding the progressive extension of the surveillance system to other Lao provinces from 2015 and onwards. In 2012 and 2013, DENV serotype identification was mainly performed for isolates collected in Vientiane Capital, which represented, respectively, 80% and 87% of the samples tested. In 2014, only 134 suspected cases were investigated. The dramatically low number of samples tested by the arbovirus surveillance network was consistent with the poor syndromic activity recorded at the country level by the National Center for Laboratory and Epidemiology in charge of the centralization of the mandatory clinical declaration system (Figure 1). Furthermore, since 2012, the laboratory-based survey seemed to follow the same dengue epidemiological profile as the national syndromic surveillance system (Figure 1). A total of 14 (10.4%) were confirmed by DENV RT-PCR in 2014 and the DENV serotype could be determined for 11 samples (78.6%) (Figure 2).

Among the cases confirmed by RT-PCR in Vientiane Capital, 85.7% and 62.4% were respectively serotyped in 2016 and in 2018 (Table S1). The proportion of samples serotyped varied from year to year (from 28.2% in 2019 to 76.6% in 2014), but the proportion of samples from Vientiane Capital remained constant over time (between 50% and 65%) (Table S1).

In 2012 and 2013, all four DENV serotypes were detected, but DENV-3 serotype was predominant (62% in 2012 and 92% in 2013) (Figure 2). This serotype gradually decreased and vanished in December 2013 [29]. Since then, only 2 DENV-3 sporadic cases were collected in Saravane in 2016 and 3 cases were detected in Vientiane Capital in 2017. In 2015, DENV-1 was at the origin of 85% of the confirmed dengue cases at the country level and this serotype continued to circulate at a low level in Lao PDR until 2019.

**Figure 3:**
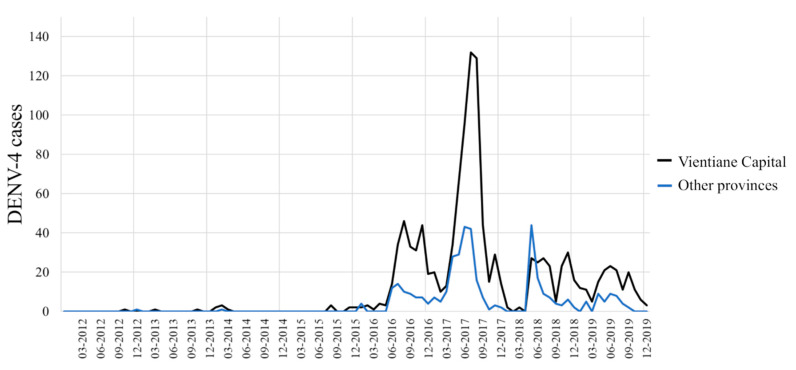
Dengue serotype 4 distribution between 2012 and 2019 per month from samples collected by the Institut Pasteur du Laos arbovirus surveillance network in Vientiane Capital and in other Lao provinces.

Between 2012 and 2015, DENV-4 samples represented less than 9% of the total cases (Figure 2) and were only recorded in the sub-urban districts of Vientiane Capital and in Vientiane Province, which are mainly rural areas. In 2014, clusters of DENV-4 cases were identified in Vientiane Capital. At the end of 2015, the dengue epidemiological profile changed in Vientiane capital with the identification of grouped cases of DENV-4 in urban districts of Vientiane Capital followed by a rapid increase of the proportion of DENV-4 cases in Vientiane Capital from 9% in 2015 to 70% and 67%, respectively, in 2016 and 2017. The outbreak peaked between June and August 2017 (Figure 3). The same trend was observed at the country level (Figure 3). In 2018, the proportion of DENV-4 samples decreased to 43% and DENV-2 became the predominant serotype at the end of the year. In 2019, at the country level, DENV-4 still represented 13% of the cases serotyped (13.2% in Vientiane Capital) with a co-circulation of DENV-1 and DENV-2 (Figure 2).

### 2.2. Epidemiological Analysis of the Patients Infected by DENV-4

**Figure 4:**
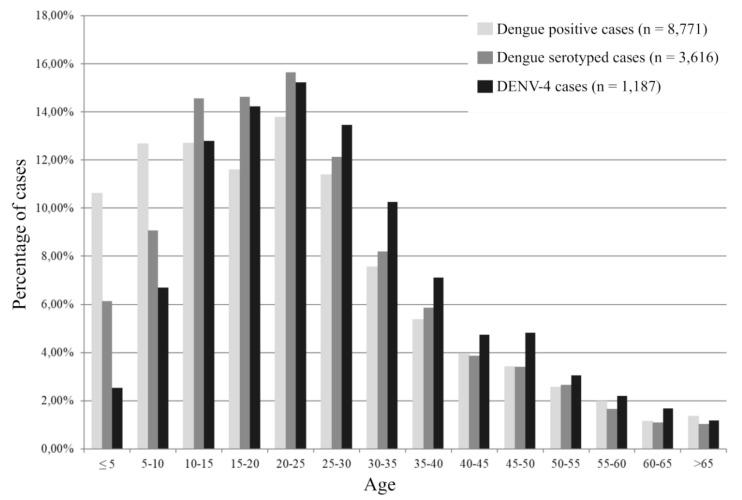
Age distribution of patients investigated by the Institut Pasteur du Laos arbovirus surveillance network.

Demographic and clinical characteristics of the patients reported by the surveillance network are presented in Table 1. Patients infected with DENV-4 serotype were significantly older (mean age 27.4 vs. 24.5, *p* < 0.0001) than patients infected with other DENV serotypes (Table 1 and Figure 4). Sex ratio, days to diagnosis, and severity were no different for DENV-4 when compared to the other serotypes.

Among the 52 DENV fatal cases investigated by the arbovirus surveillance network between 2012 and 2019, 9 were infected by DENV-4. These nine cases were identified between 2016 and 2019. In Vientiane Capital, four cases were detected in September 2016, August 2017, January 2019, and December 2019. In Attapeu province, 2 DENV-4 fatal cases were identified in July and September 2017. Two fatal cases were also recorded in June 2018 in Xayaboury and Champassak provinces and 1 case in Saravane province in April 2019 (Figure S1).

### 2.3. Phylogenetic Analysis of DENV-4

**Figure 5:**
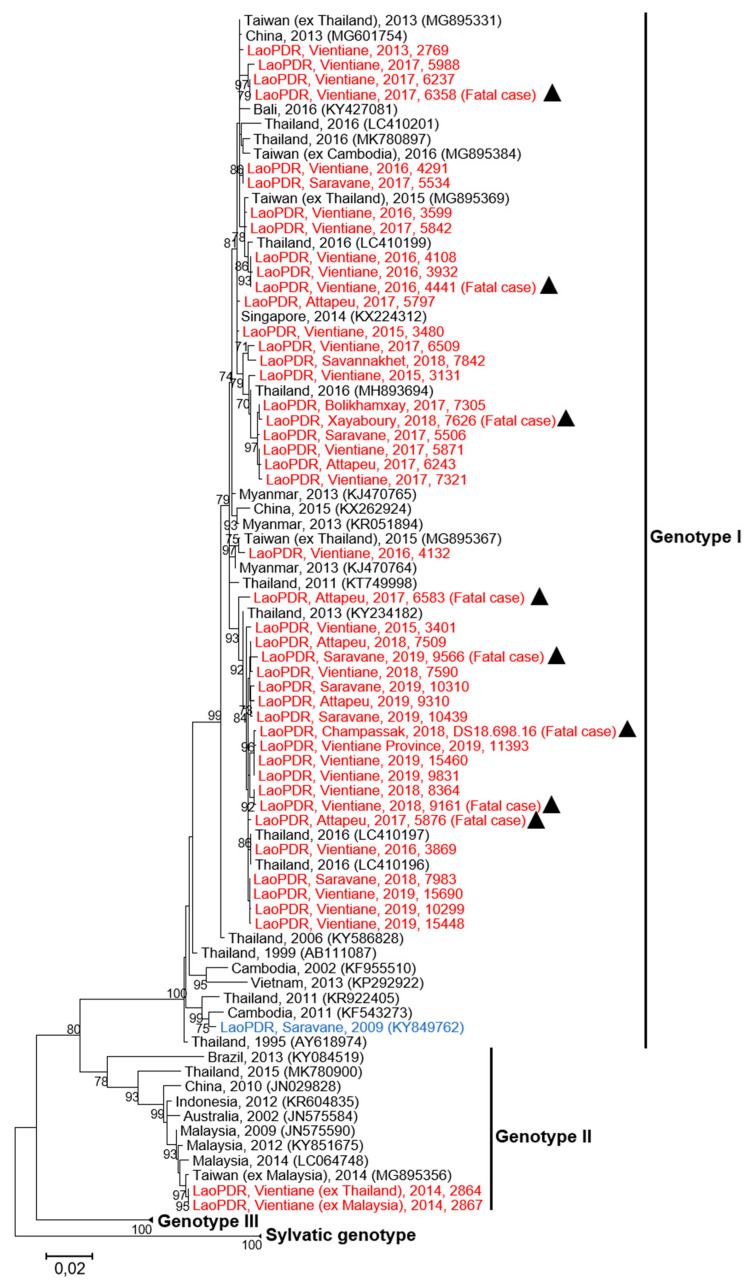
Maximum likelihood phylogenetic tree of DENV-4 sequences from Lao PDR. The tree was constructed on the envelope protein gene (1485 nt). Only the bootstrap values > 70 are shown. Scale bar indicates the nucleotide substitution per site. The Lao strains sequenced in this study are indicated in red and the triangles highlight the strains from fatal cases. The Lao strain previously described is indicated in blue. Black triangles identify isolates obtained from fatal cases.

A panel of 45 samples, i.e., plasma (*n* = 29) or cell supernatant (*n* = 16), from DENV-4 cases collected between 2013 and 2019, were investigated for the phylogenetic analysis (Table 2). This panel included samples from different Lao provinces, cases imported from foreign Asian countries, patients with different degrees of dengue severity, and fatal cases. The sequence analysis based on a fragment spanning 1485 nucleotides of the envelop gene revealed that DENV-4 isolates grouped in two different genotypes (i.e., Genotype I and Genotype II) (Figure 5).

Genotype II was identified in samples from European tourists traveling in Southeast Asia (Thailand and Malaysia) in 2014 and who developed a dengue-like syndrome during their stay in Vientiane. These isolates clustered with DENV-4 strains identified in Malaysia in 2009 and 2014 (>99% identity) (Figure 5) [32].

All autochthonous DENV-4 sequences belonged to the Genotype I. Among the Genotype I, the Lao strains isolated between 2013 and 2019 displayed a maximum nucleotide distance of 2.7% from each other. Interestingly, our series of isolates showed 3.1% to 4.1% of divergence with a Lao strain (KY849762) isolated in 2009 in Saravane province (Figure 5) [30]. When compared to DENV-4 reference strains collected in other Asian countries from 2011 to 2016, less than 2.5% of nucleotide divergence was found with the Lao DENV-4 isolates (Figure 5). In this study, eight DENV-4 isolates from fatal cases could be analyzed. These isolates belonged to the Genotype I but segregated in different phylogenetic clusters formed by the different Lao isolates characterized since 2013 (Figure 5). Globally, the phylogenetic analysis revealed the active circulation of DENV-4 Genotype I since at least 2013 in Lao PDR. Furthermore, several clusters were detected and could emphasize several introduction events in the country from neighboring countries such as Thailand (Figure 5).

### 2.4. Entomological Survey on the Aedes sp. Vectors in Vientiane Capital

**Figure 6:**
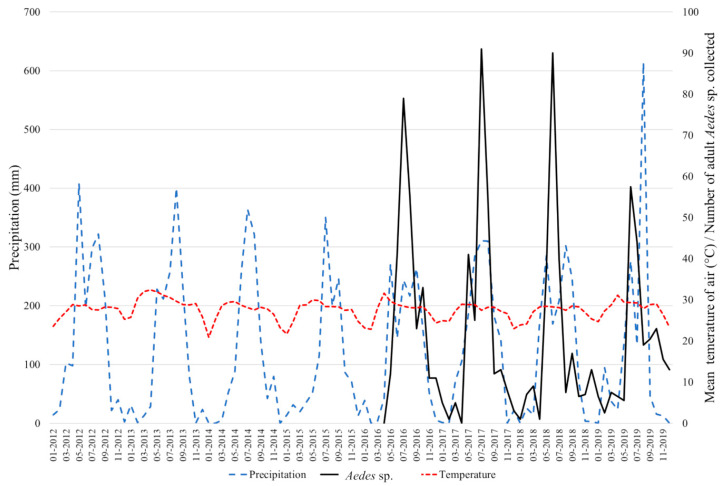
Monthly rainfall (mm; in blue) and mean temperature in the air (°C; in red) recorded between 2012 and 2019 in Vientiane Capital (17°58′12″ N, 102°34′14″ E). Number of *Aedes aegypti* and *Aedes albopictus* adult mosquitoes collected monthly between 2016 and 2019 in Vientiane Capital (in black).

The results of the abundance of the adult dengue vectors collected between 2016 and 2019 are presented in Figure 6. A total of 1276 specimens were collected and *Aedes aegypti* was the most abundant species, representing more than 86% of the total. *Aedes albopictus* represented 14% of the total. The percentage of *Ae. albopictus* varied between 3.2% in 2018 to 57.8% in 2016 when the study only started in May. In 2017 and 2019, the percentages were 12.6% and 9.9%, respectively. Every year, the vectors were more abundant during the rainy season between May and August, but the mosquito populations remained throughout the dry season albeit at a low level. Of note, no collections were made during the month of April; and in 2016, the collection started in May only.

## 3. Discussion

Since the first report on dengue in Lao PDR, limited data have been published on DENV serotypes/genotypes circulating at the country level [22]. Nevertheless, recent studies suggest a complex and dynamic dengue virus circulation countrywide, but without reference to DENV-4 [22,28,29,30]. This study is a first step in filling this gap by providing genetic and epidemiologic information on the first DENV-4 epidemic recorded in Lao PDR. The progressive increase of DENV-4 burden occurred in a context of the co-circulation of different DENV serotypes previously described and maintained over the eight years of surveillance (i.e., 2012–2019) [29,30,31]. Since 2009, DENV-4 circulated sporadically in the country [29,30]. Sporadic cases or small clusters of grouped cases were observed in Vientiane Capital between February 2014 and August 2015. Subsequently, the proportion of DENV-4 cases gradually increased among the samples tested by the arbovirus surveillance network in Vientiane Capital and became predominant in 2016 at the country level until October 2018. Since then, DENV-2 has overcome DENV-4 in Vientiane Capital and at the country level [33]. However, DENV-4 still circulated at a significant level in 2019 (13%; Figure 2). These data reveal a circulation of DENV-4 during at least six years (2014–2019) in Lao PDR and could be correlated with an insufficient immunity protection of the Lao population against this serotype as suggested by the impact of DENV-4 in the different age groups.

This study highlighted the significant difference in the age of the patients infected with DENV. The syndromic patients infected with DENV-4 were significantly older than the patients infected with other serotypes. The Lao population is young (~40% under 20 years old) (WHO and www.lsb.gov.la) and this data emphasize the risk of dengue emergence in Lao PDR due to a high percentage of the population immunologically naive for DENV. In this study, the mean age (27.4) of the patients infected by DENV-4 appeared to be consistent with studies from other DENV-endemic countries [34,35]. However, the fact that syndromic DENV-4 patients were older could suggest the recent emergence of this serotype and/or a low DENV-4 circulation in Lao PDR at least since 2009 [30]. These findings demonstrate that a large proportion of the Lao population is susceptible to DENV-4 and highlights the need to study the immune status of populations living in dengue-endemic countries in order to predict and prevent future DENV outbreaks as previously done in Lao PDR [22] and in Asia in general [36,37,38]. Furthermore, this study highlights the need to perform serotype-specific studies of the immune status of the population.

Here, we describe the pre-epidemic circulation of DENV-4 in Lao PDR and the specific features of this serotype during the outbreak in Vientiane Capital and at the country level in the context of DENV serotypes co-circulation. Clusters of DENV-4 infections were identified sporadically several years before the outbreak started. Indeed, serotype-specific surveillance networks are able to finely monitor the profile of DENV circulating and detect early warning signals. Strengthening the prediction capacity of DENV outbreaks by combining syndromic surveillance systems with a laboratory surveillance system has been demonstrated in Lao PDR ([30]; present study). Even if a systematic exhaustive investigation of suspected cases was clearly unrealistic, an appropriate sample size allowed for the drawing of an accurate picture of DENV serotypes’ history in a country over the last eight years. In this way, the actual disease burden of dengue could be calculated, including at the serotype level. The amplitude over time and the burden by serotype and by age groups could be used as proxies to estimate the level of herd immunity in the Lao population. Interestingly, the local lab-based surveillance system in Vientiane city followed the trends of the syndromic surveillance data. Based on this approach, mathematical models could be developed to determine parameters including costs of long-term sustainable, combined surveillance systems.

The disease severity of dengue fever was not associated with the predominance of DENV-4 in Lao PDR. Indeed, no significant difference in the disease severity was found between the DENV serotypes in the confirmed samples collected by the arbovirus surveillance network. This finding is in agreement with previous observations, such as during a DENV-4 outbreak in Brazil, which failed to show any link between this serotype and dengue severe symptoms [20]. Furthermore, in Lao PDR, the fatal cases due to a DENV-4 infection were recorded during the entire outbreak and no link could be established with the number of positive DENV-4 cases. Indeed, in our study, the same number of fatal cases was recorded in 2017 and 2019, respectively, during the peak of the outbreak and at the end of the outbreak. Moreover, dengue fatal cases were observed during the active DENV transmission period, as well as when dengue cases were recorded at a low level.

Previous phylogenetic studies showed the recent circulation of two DENV-4 genotypes (Genotype I and II) in Southeast Asia [18,32]. Both genotypes were detected in Lao PDR, but in two different and independent types of patients, i.e., Lao native autochthonous cases and imported cases by foreigners. All autochthonous cases recorded at the country level investigated here belonged to DENV-4 Genotype I. The first evidence of the presence of DENV-4 Genotype I in Lao PDR came from a strain that was first identified in Saravane Province isolated in 2009 [30]. Our serial investigation suggests that this genotype has spread and circulated at least since 2013 at the country level. The genetic difference (2.7% of nucleotide divergence) between the former strain and the recent isolates could be the result of several introductions of DENV-4 Genotype I in Lao PDR since 2009 from different Asian countries. The composition of the different clusters and the circulation of the Lao isolates over several years are a signature of successful consecutive emergences of those independent introduction events. DENV-4 Genotype II was only detected in two imported cases from Thailand/Malaysia in 2014 (Figure 5). Interestingly, the predominant circulation of the DENV-4 Genotype I in Lao PDR contrasted with some results obtained in South East Asia. Indeed, in the Philippines, a genotype turnover from Genotype I to Genotype II was observed before 2013, and this seemed to match with a high DENV-4 circulation in the country between 2012 and 2016 [39]. The results found in the Philippines were consistent with those from Malaysia in 2001 that showed the circulation of the DENV-4 Genotype II [40]. However, both introductions of DENV-4 Genotype II were recorded in February 2014, the dry season in Lao PDR (October–April) and a decrease of vector density. This context, associated with the abnormal lack of rainfalls until August 2015, could explain the non-emergence of DENV-4 Genotype II in Vientiane Capital. However, these dramatically unfavorable conditions for DENV transmission did not hamper the maintenance of a sufficient inoculum of a mixt population of DENV-4 Genotype I isolates at the origin of the rapid spread-off since September 2015. Hence, the genotype selection could impact the epidemiology of DENV emergence and expansion of the virus. Indeed, as previously described in India, China, or the WHO Pacific region, a DENV genotype switch could increase the risk of dengue outbreak [41,42,43]. This selection of DENV genotype could be influenced by several factors, such as serotype-specific herd immunity, viremia titer in humans, and the ability of local mosquito populations, such as *Aedes aegypti*, to transmit the virus under the influence of environmental and climatic conditions [44,45,46,47,48,49]. These factors need to be investigated for DENV serotypes and genotypes, which circulated in a specific geographical context to improve the knowledge on DENV epidemiology and to prevent DENV outbreaks [50].

The entomological results demonstrate the presence of both *Ae. aegypti* and *Ae. albopictus* in Vientiane Capital with an increase of vector density during the rainfall season as previously described in Asia. The dynamic of DENV transmission in human population follows the trend of the dynamic of *Aedes* vectors [51]. Moreover, predominance of *Ae. aegypti* was highlighted since 2016 in Vientiane Capital. Vector competence of *Ae. aegypti* from Lao PDR was investigated for DENV-1 and showed that 50% of the females orally exposed to the virus could transmit DENV-1 [52]. These data indicate a high efficiency of transmission of this serotype by *Ae. aegypti* from Lao PDR compared to other *Ae. aegypti* populations from the Pacific region (3–37% transmission efficiency), from French Guiana (<10% transmission efficiency), and from Florida (33% transmission efficiency) in the USA [53,54,55]. DENV transmission ability appeared to be a specific interaction between the mosquito population and a virus strain [49,56]. Vazeille et al. [54] suggested a possible competition between serotypes at the midgut level in co-infected mosquitoes leading to a drastically different transmission potential and, in this case, favoring the competitive displacement of DENV-1 by DENV-4. Thus, the ability of Lao vectors for DENV-4 must be investigated to determine how this factor could influence the onset of new dengue epidemics in Lao PDR.

DENV emergence and spread are complex mechanisms, especially in endemic countries like Lao PDR. This study shows the need to combine results obtained by the DENV laboratory-based surveillance system and epidemiologic studies, combined with entomological data, along with more research oriented approaches, such as virus genetic, viral kinetic, or virus/vector interaction findings, to determine the impact of these factors on DENV transmission in each specific geographical context.

## 4. Materials and Methods

### 4.1. Human Samples Collection

In 2012, an arbovirus surveillance system was set up and coordinated by the Institut Pasteur du Laos. It was first implemented in Vientiane Capital and then progressively extended to hospitals in 18 provinces, with Vientiane Capital’s hospital network being the most active. From 2012 to 2019, human samples were collected through this surveillance hospital network. Suspected dengue fever cases were defined according to the WHO’s definition (fever onset ≥ 38 °C for less than 7 days with at least one of the following accompanying symptoms: headache; myalgia; arthralgia; retro-orbital pain; digestive troubles or hemorrhaging) [29]. After obtaining informed consent, clinicians filled a standardized clinical report form (CRF). Cases were classified according to the WHO criteria (1997), defining the severity as Dengue fever (DF), Dengue hemorrhagic fever (DHF), or Dengue shock syndrome (DSS). Venous blood samples (5 mL) were taken from patients. Samples were stored at 4 °C during transportation to the Institut Pasteur du Laos for analysis and serotypes and genotypes investigation.

### 4.2. Ethical Statement

Ethics approval was obtained from the Lao National Ethics Committee for Health Research (N°2018.116). Oral and written informed consent were obtained from all participants, or a parent or legal guardian.

### 4.3. Dengue Virus Screening

Samples collected by the arbovirus surveillance system were screened by RT-PCR as previously described [29] with a pan-dengue real-time RT-PCR [57] and the DENV serotypes were determined with specific real-time RT-PCR [58].

### 4.4. Gene E Sequencing Analysis

Viral genomic RNA was extracted from 45 human plasma using a Nucleospin DX or Nucleospin 96 core kit purification kit (Macherey-Nagel) according to the manufacturer’s instructions (Table 2).

Gene E sequencing (1485nt) was performed using primers FGT1, FGT2, and F3 (Table 3). First Stand cDNA were generated using a Maxima H Minus First Stand cDNA Synthesis kit (Thermo Scientific, Waltham, MA, USA) and the PCR was performed using a Phusion Flash High-Fidelity PCR Master Kit (New England Biolabs^®^ Inc, Waltham, MA, USA). Amplified fragments were purified using ExoSAP-IT^TM^ PCR Product Cleanup Reagent (Thermo Fisher Scientific, Waltham, MA, USA). Purified fragments were sequenced using a BigDye Terminator v3.1 Cycle sequencing kit (Applied Biosystem, Waltham, MA, USA) on a Genetic Analyzer 3500xL (Applied Biosystem, Waltham, MA, USA).

Sequences were analyzed using Chromas software (www.technelysium.com.au) and aligned with the multiple sequence alignment software Clustal W integrated in BioEdit version 7.0.5.3 software (Manchester, United Kingdom) [59,60]. For the phylogenetic analysis, a maximum likelihood tree was constructed using MEGA version 7 (www.megasoftware.net), with a kimura-2 parameter model with a bootstrap of 1000 replication [61] as previously described for dengue virus phylogenetic analysis [29,43,62,63,64]).

### 4.5. Epidemiological Analysis

Demographic and clinical data were extracted from the surveillance network’s de-identified database. Continuous variables were summarized using mean and standard deviation (SD), and categorical variables were summarized using frequencies and percentages. Continuous data were compared using parametric Student test and rates using the Chi-square and Fisher exact test. Statistical significance was set at the 5% level. Analyses were conducted using STATA software (version 14.0; StatCorp LP, College Station, TX, USA).

### 4.6. Mosquito Surveillance

Five mosquito sentinel sites in five different villages of Vientiane Capital were chosen for the mosquito surveillance and the GPS locations are presented in Table 4. The vectors abundance study took place during both dry and rainy seasons between 2016 and 2019. BG sentinel traps^®^ (Biogents, Regensburg, Germany) were used to follow the dynamics of mosquito population abundance in the city (4 traps per locations, except at Kao-Gnot with two traps). After every weekly collection, the adult mosquitoes were brought back to the laboratory for morphological identification.

## Figures and Tables

**Table 1:** Demographic and clinical characteristics of the 15,152 patients investigated by the Institut Pasteurs du Laos arbovirus surveillance system between 2012 and 2019.

	All Samples (*n* = 15,152)	Dengue Positive ^a^ (*n* = 8771)	Sample Serotyped (DENV-1 to DENV-4) (*n* = 3616)	DENV-4 (*n* = 1187)
Mean age (SD)	23.5 (15.7) *n* = 15,062	23.9 (14.4) *n* = 8718	24.5 (14.5) *n* = 3589	27.4 (14.6) *n* = 1181
Sex				
Female (%)	6197 (40.9)	3775 (43.0)	1526 (42.3)	620 (52.2)
Male (%)	5945 (39.2)	3243 (39.3)	1403 (38.8)	557 (46.9)
Unknown (%) ^†^	3010 (19.9)	1550 (17.7)	682 (18.9)	10 (0.8)
Mean number of days of fever (SD)	3.8 (2.4)	3.6 (1.8)	3.4 (1.8)	3.6 (1.7)
Clinical diagnosis ^b^				
DF (%)	11,910 (78.6)	7036 (80.2)	2868 (79.4)	1154 (97.2)
DHF (%)	213 (1.4)	168 (1.9)	49 (1.4)	13 (1.1)
DSS (%)	27 (0.2)	23 (0.3)	17 (0.5)	9 (0.8)
Unknown (%) ^†^	3002 (19.8)	1541 (17.6)	677 (18.8)	11 (0.9)

Note: ^a^ Dengue-positive: RT-PCR and or NS1-positive. ^b^ Data concerning clinical diagnosis were collected from 2015 onwards. DF: Dengue fever, DHF: Dengue hemorrhagic fever, DSS: Dengue shock syndrome. ^†^ No clinical data.

**Table 2:** References of Lao DENV-4 isolates.

Sample Identification	Years of Collection	Isolation Source	Genbank Number
LaoPDR-Vientiane, 2013-2769	2013	Cell supernatant	MT122852
LaoPDR-Vientiane (ex Thailand), 2014-2864	2014	Cell supernatant	MT122853
LaoPDR-Vientiane (ex Malaysia), 2014-2867	2014	Cell supernatant	MT122854
LaoPDR-Vientiane, 2015–3131	2015	Cell supernatant	MT122855
LaoPDR-Vientiane, 2015–3401	2015	Cell supernatant	MT122856
LaoPDR-Vientiane, 2015–3480	2015	Cell supernatant	MT122857
LaoPDR-Vientiane, 2016–3599	2016	Cell supernatant	MT122858
LaoPDR-Vientiane, 2016–3869	2016	Cell supernatant	MT122859
LaoPDR-Vientiane, 2016–3932	2016	Cell supernatant	MT122860
LaoPDR-Vientiane, 2016–4108	2016	Cell supernatant	MT122861
LaoPDR-Vientiane, 2016–4132	2016	Cell supernatant	MT122862
LaoPDR-Vientiane, 2016–4291	2016	Cell supernatant	MT122863
LaoPDR-Vientiane, 2016–4441 (Fatal case)	2016	Plasma	MT122864
LaoPDR-Saravane, 2017–5506	2017	Plasma	MT122865
LaoPDR-Saravane, 2017–5534	2017	Cell supernatant	MT122866
LaoPDR-Attapeu, 2017–5797	2017	Cell supernatant	MT122867
LaoPDR-Vientiane, 2017–5842	2017	Cell supernatant	MT122868
LaoPDR-Vientiane, 2017–5871	2017	Plasma	MT122869
LaoPDR-Attapeu, 2017–5876 (Fatal case)	2017	Plasma	MT122870
LaoPDR-Vientiane, 2017–5988	2017	Plasma	MT122871
LaoPDR-Vientiane, 2017–6237	2017	Plasma	MT122872
LaoPDR-Attapeu, 2017–6243	2017	Plasma	MT122873
LaoPDR-Vientiane, 2017–6358 (Fatal case)	2017	Plasma	MT122874
LaoPDR-Attapeu, 2017–6583 (Fatal case)	2017	Plasma	MT122875
LaoPDR-Vientiane, 2017–6509	2017	Plasma	MT122876
LaoPDR-Bolikhamxay, 2017–7305	2017	Plasma	MT122877
LaoPDR-Vientiane, 2017–7321	2017	Plasma	MT122878
LaoPDR-Attapeu, 2018–7509	2018	Plasma	MT122879
LaoPDR-Vientiane, 2018–7590	2018	Plasma	MT122880
LaoPDR-Xayaboury, 2018–7626 (Fatal case)	2018	Plasma	MT122881
LaoPDR-Savannakhet, 2018–7842	2018	Plasma	MT122882
LaoPDR-Saravane, 2018–7983	2018	Plasma	MT122883
LaoPDR-Vientiane-2018–8364	2018	Plasma	MT122884
LaoPDR-Vientiane, 2018–9161 (Fatal case)	2018	Plasma	MT122885
LaoPDR-Champassak, 2018-DS18-698-16 (Fatal case)	2018	Cell supernatant	MT122886
LaoPDR-Attapeu, 2019–9310	2019	Plasma	MT122887
LaoPDR-Saravane, 2019–9566 (Fatal case)	2019	Plasma	MT122888
LaoPDR-Vientiane, 2019–9831	2019	Plasma	MT122889
LaoPDR-Vientiane, 2019–10299	2019	Plasma	MT122890
LaoPDR-Saravane, 2019–10310	2019	Plasma	MT122891
LaoPDR-Saravane, 2019–10439	2019	Plasma	MT122892
LaoPDR-VientianeProvince, 2019–11393	2019	Plasma	MT122893
LaoPDR-Vientiane, 2019–15448	2019	Plasma	MT122894
LaoPDR-Vientiane, 2019–15460	2019	Plasma	MT122895
LaoPDR-Vientiane, 2019–15690	2019	Plasma	MT122896

**Table 3:** List and positions of primers used for DENV-4 complete envelope gene RT-PCR and sequencing.

Fragment	Forward	Genome Position	Reverse	Genome Position
FGT1	^5′^CAT-TCA-GGA-ATG-GGA-TTG-GA^3′^	732–751	^5′^ACA-GTC-CAC-AAT-GGA-GAY-AC^3′^	1362–1381
FGT2	^5′^AGG-AGG-AGT-TGT-GAC-ATG^3′^	1268–1285	^5′^TTG-GGC-GCA-TCA-TCA-CAT^3′^	1981–1998
F3	^5′^GAG-ATG-GCA-GAA-ACW-CAG-C^3′^	1869–1887	^5′^TTA-GAT-CAA-CCA-CGA-GGC-T^3′^	2593–2611

The genome positions are given according to the dengue virus serotype 4 reference genome (GenBank: NC_002640).

**Table 4:** Mosquito collection locations in Vientiane Capital.

District	Village	Latitude	Longitude
Sittattanak	Donkoy	17.562677	102.390768
Sittattanak	Kao-Gnot	17.962684	102.615035
Xaysettha	Sengsavang	17.995816	102.664895
Xaithany	Sivilay	18.003705	102.380003
Sittattanak	Saphanthong Tai	17.949470	102.628487

## Supplementary Materials

The following are available online at https://www.mdpi.com/2076-0817/9/9/728/s1, Figure S1: Map of Lao PDR, Table S1: Geographic origins of the DENV samples serotyped by the Institut Pasteur du Laos arbovirus surveillance system.
