# Supplementary material for: Trends of the Dengue Serotype-4 Circulation with Epidemiological, Phylogenetic, and Entomological Insights in Lao PDR between 2015 and 2019
Source: Pathogens. 2020 Sep 3;9(9):728. doi: 10.3390/pathogens9090728 (PMC7557816; doi:10.3390/pathogens9090728)
Supplement: Supplementary file 1 [file pathogens-09-00728-s001.pdf]

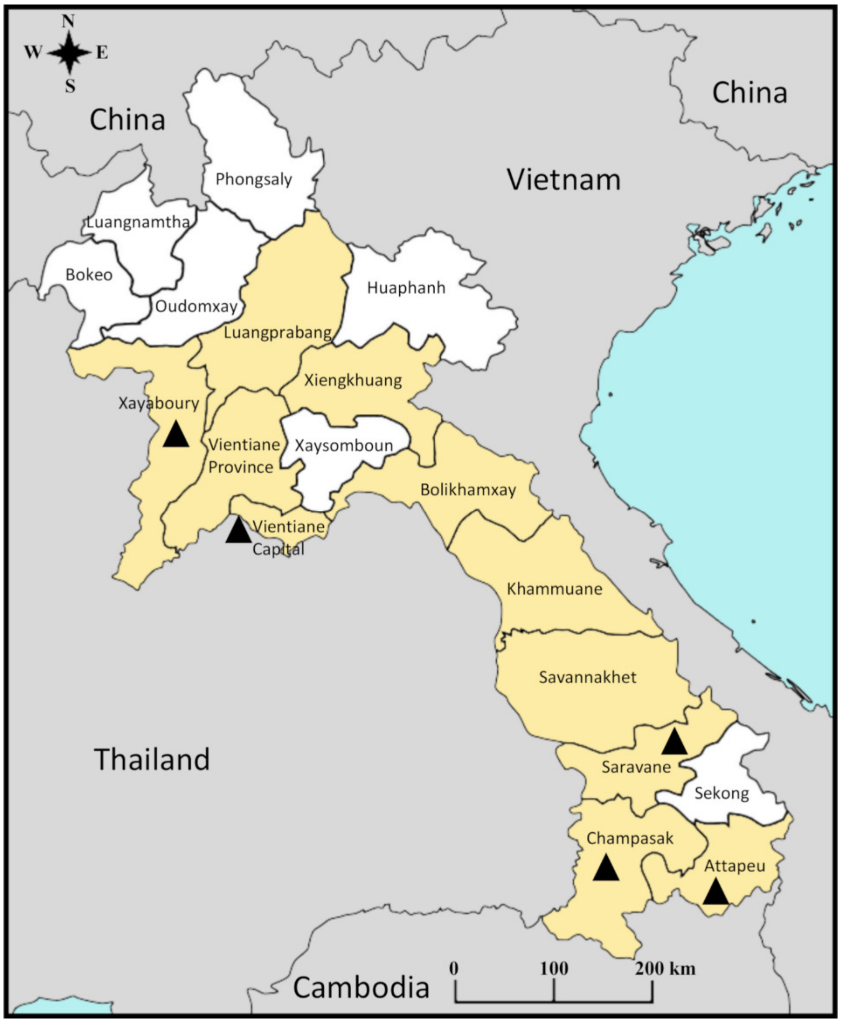

Figure S1. Map of Lao PDR. Provinces with DENV-4 reported cases between 2012 and 2019 are in yellow. Triangles indicate the localization of DENV-4 fatal cases.

Table S1. Geographic origins of the DENV samples serotyped by the Institut Pasteur du Laos arbovirus surveillance system.

**Province**	**2012**	**2013**	**2014**	**2015**	**2016**	**2017**	**2018**	**2019**	**Total**
Vientiane capital	105	466	8	65	286	644	290	869	2,733
Attapeu	0	0	0	25	6	69	111	62	273
Bokeo	0	2	0	0	0	0	0	0	2
Bolikhamxay	0	3	0	0	0	0	2	9	14
Champassak	0	1	0	0	0	0	3	41	45
Huaphanh	0	1	0	0	0	0	0	0	1
Khammuane	0	1	0	0	0	3	0	1	5
Luangprabang	1	0	0	0	0	0	2	15	18
Oudomexay	1	0	0	0	0	0	0	1	2
Saravane	0	0	0	0	42	150	29	102	323
Savannakhet	0	0	0	0	0	0	19	25	44
Vientiane province	8	38	0	0	0	4	9	37	96
Xayaboury	0	1	0	0	0	0	1	2	4
Xaysomboun	0	0	0	0	0	0	0	1	1
Xiengkhuang	0	0	0	0	0	1	0	12	13
Unknown	16	23	3	0	0	0	0	0	42
Total	131	536	11	90	334	871	466	1,177	3,616
